# Health system reforms in five sub-Saharan African countries that experienced major armed conflicts (wars) during 1990–2015: a literature review

**DOI:** 10.1080/16549716.2018.1517931

**Published:** 2018-10-01

**Authors:** Chol Chol, Joel Negin, Alberto Garcia-Basteiro, Tesfay Gebregzabher Gebrehiwot, Berhane Debru, Maria Chimpolo, Kingsley Agho, Robert G Cumming, Seye Abimbola

**Affiliations:** a School of Public Health, Faculty of Medicine and Health, the University of Sydney, Sydney, Australia; b Centro de Investigação em Saúde de Manhiça (CISM), Maputo, Mozambique; c School of Public Health, Mekelle University, Mekelle, Ethiopia; d Research and Human Resource Development, Ministry of Health, Asmara, The State of Eritrea; e Faculdade de Medicina, Universidade Agostinho Neto, Luanda, Angola; f School of Science and Health, Western Sydney University, Sydney, Australia

**Keywords:** Angola, armed conflict, community healthcare workers, decentralisation, Eritrea, Ethiopia, fragile states, health-financing system, Mozambique, Rwanda, war

## Abstract

**Background**: Sub-Saharan Africa (SSA) has had more major armed conflicts (wars) in the past two decades – including 13 wars during 1990–2015 – than any other part of the world, and this has had an adverse effect on health systems in the region.

**Objective**: To understand the best health system practices in five SSA countries that experienced wars during 1990–2015, and yet managed to achieve a maternal mortality reduction – equal to or greater than 50% during the same period – according to the Maternal Mortality Estimation Inter-Agency Group (MMEIG). Maternal mortality is a death of a woman during pregnancy, or within 42 days after childbirth – measured as maternal mortality ratio (MMR) per 100,000 live births.

**Design**: We conducted a selective literature review based on a framework that drew upon the World Health Organisation’s (WHO) six health system building blocks. We searched seven databases, Google Scholar as well as conducting a manual search of sources in articles’ reference lists – restricting our search to articles published in English. We searched for terms related to maternal healthcare, the WHO six health system building blocks, and names of the five countries.

**Results**: Our study showed three general health system reforms across all five countries that could explain MMR reduction: health systems decentralisation, the innovation related to the WHO workforce health system building block such as training of community healthcare workers, and governments-financing reforms.

**Conclusion**: Restoring health systems after disasters is an urgent concern, especially in countries that have experienced wars. Our findings provide insight from five war-affected SSA countries which could inform policy. However, since few studies have been conducted concerning this topic, our findings require further research to inform policy, and to help countries rebuild and maintain their health systems resilience.

## Background

War-affected healthcare systems in sub-Saharan Africa has adopted some reforms to rebuild their healthcare systems, and to mitigate the aftermath of disasters such as wars. These reforms are essential since SSA is the region of the world that has had the highest number of armed conflicts (including major armed conflicts (wars) with at least 1,000 battle-related direct deaths per year) in the past two decades [1–3]. In addition to causing health systems destruction, wars have disproportionately affected women’s and children’s health and severely impaired the provision of maternal health services [4–7].

The high prevalence of armed conflicts in SSA and their detrimental effect on health systems’ provision of maternal health services could be one of the reasons why the region also has the highest numbers of maternal deaths worldwide – a death of a woman while pregnant or within 42 days after childbirth [8]. The 2015 Maternal Mortality Estimation Inter-Agency Group (MMEIG) showed that SSA accounted for 66% of all maternal deaths worldwide [8]. Besides, 19 of the 49 SSA countries have an extremely high maternal mortality – measured as the maternal mortality ratio (MMR) – equal to or greater than 500 deaths per 100,000 live births [8].

Despite SSA’s poor record in reducing its MMR during 1990–2015, there have been some notable successes to reduce MMR. For example, amongst 14 SSA countries that achieved MMR reductions equal to or greater than 50%, five of those countries managed to achieve a remarkable MMR reduction despite their long history of wars. These countries – listed alphabetically – are Angola, Eritrea, Ethiopia, Mozambique, and Rwanda. The wars experienced by these countries include the longest recent war in SSA – 50 years – between Eritrea and Ethiopia (1961–1991; 1998–2000; 2004), the brutal genocide in Rwanda (1994) when one million people are estimated to have been killed, and the long wars in the two the Portuguese former colonies, Angola (1975–2002) and Mozambique (1977–1992).

While some studies have examined health system reforms in low- and middle-income countries [9–14], to our knowledge, no study has examined the relationship between successful MMR reduction and health system reforms in relation to the war in SSA. To better understand this relationship in such contexts, we conducted a selective literature review of health system reforms in the five war-affected SSA countries during 1990–2015, using a framework that includes the WHO six health system building blocks [2,3,15]. We aimed to understand these countries' best health system practices which enabled them to achieve an MMR reduction equal to or greater than 50% despite the effect of war.

## Methods

This study investigated war-affected SSA countries that successfully reduced maternal mortality. Therefore, we chose to restrict our study design to these five countries, rather than to extend it to all war-affected countries in SSA.

### Selection of study countries

We used two criteria for the selection of the SSA countries: (1) those that experienced major armed conflicts (wars) during 1990–2015 [2]; (2) those that achieved ≥ 50% MMR reduction during 1990–2015 – as MMR reduction is indicative of the robustness of health systems performance [16,17].

According to the Uppsala Conflict Data Program’s definition of war (major armed conflicts with at least 1,000 battle-related direct deaths per year), during the period 1990–2015, 13 SSA countries experienced wars [2,3]. These countries with their MMRs per 100,000 live births in 2015 (alphabetically) are: Angola (477), Burundi (712), Central African Republic (CAR) (882), Democratic Republic of Congo (DRC) (693), Eritrea (501), Ethiopia (353), Liberia (725), Mozambique (489), Rwanda (290), Sierra Leone (1,360), Somalia (732), South Sudan (789), and Sudan (311) [2,8]. Sudan and South Sudan were excluded because both countries shared the same MMR data until the latter gained independence in 2011. Amongst the 11 remaining countries, we identified five countries (alphabetically) that had achieved an MMR reduction equal to or greater than ≥ 50% during 1990–2015: Angola, Eritrea, Ethiopia, Mozambique, and Rwanda.

### Data extraction and analysis

**Figure 1.**
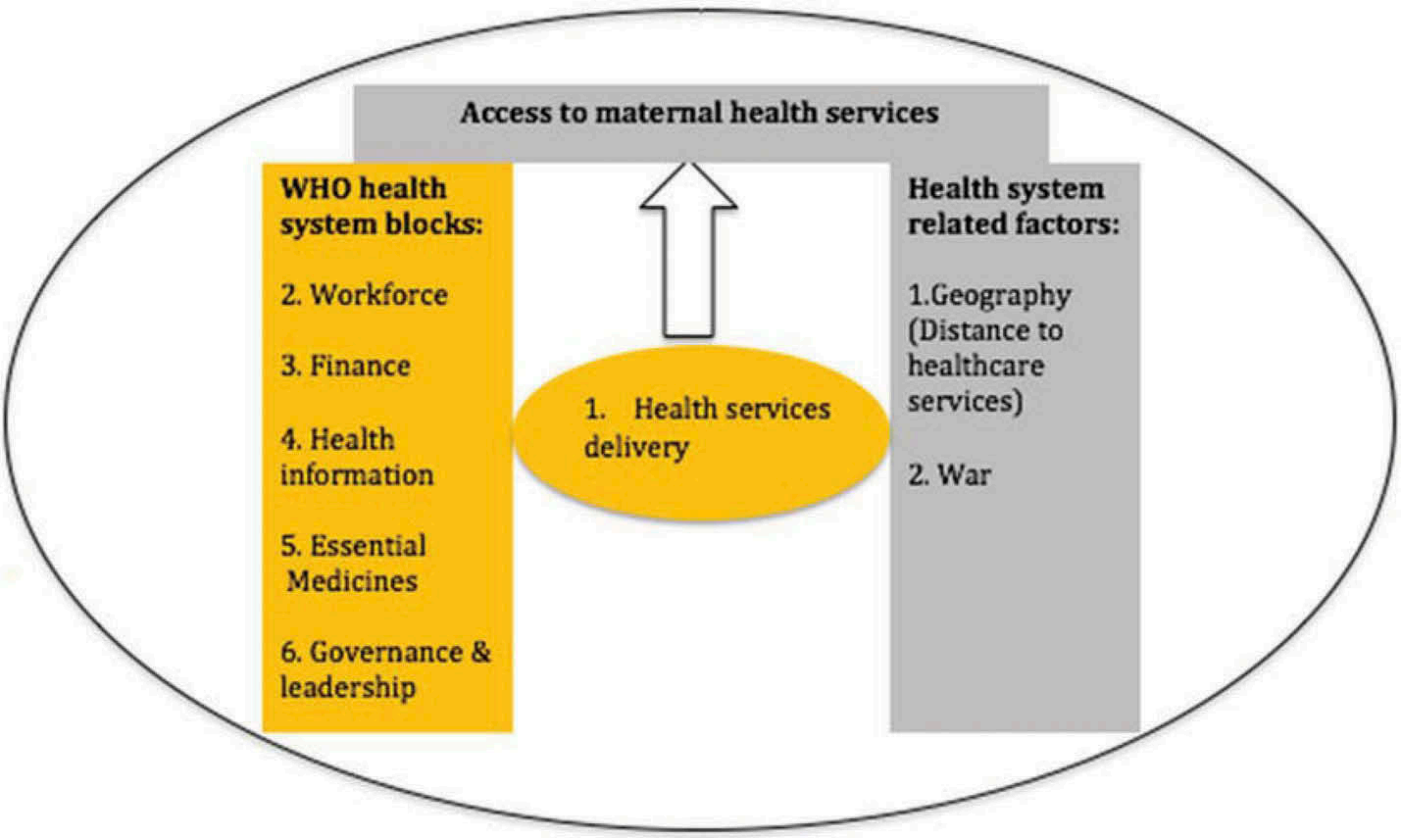
Factors affecting access to maternal health services in war affected sub-Saharan African countries, 1990–2015. Adapted from the WHO six health system blocks (2010).

**Figure 2.**
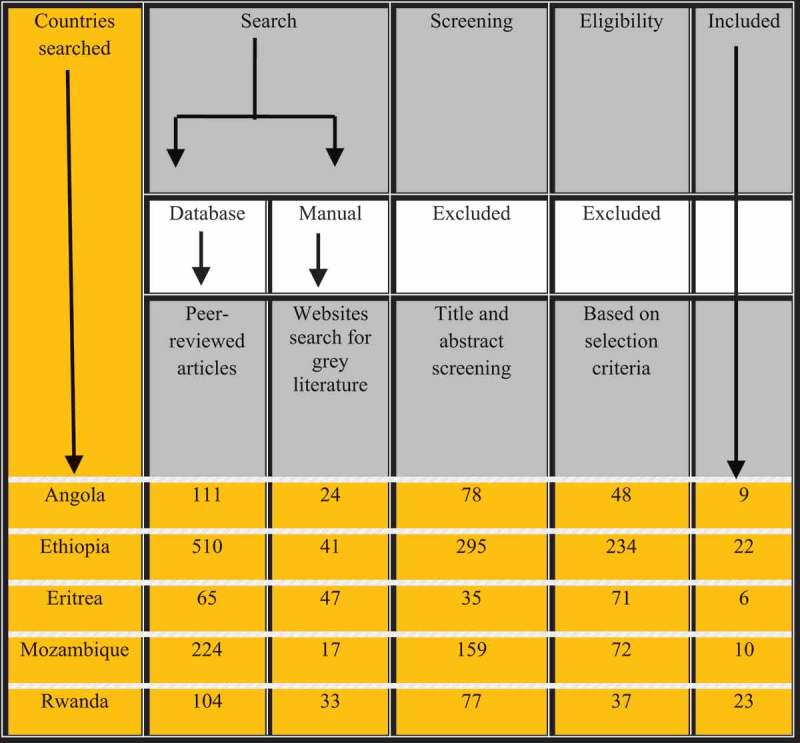
Studies inclusion criteria for the literature search and the number and type of papers included.

We aimed to identify the common health system reforms across the five countries as follows. First, for each country categorised under the headings listed in the following framework (Figure 1), articles that met the inclusion criteria (Figure 2) were extracted. Second, grey literature findings were combined with results from peer-reviewed studies for cross-validation. Third, all articles were analysed to identify any patterns across all five countries. All the previous steps were conducted by the first author (CC) and the inclusion criteria were discussed with three public health experts – RGC, JN, and SA. Finally, the results were shared for cross-validation of the findings with country-specific experts based in each of the five countries, to review and contribute further insights into the context of each country.

Because our research aimed to explore a wide range of factors that could explain health systems strengthening in five SSA countries that experienced wars during 1990–2015, we conducted a selective literature search as used by Gabrysch and Campbell [18]. We did not conduct a systematic review because such analyses are better suited for investigating defined and specific questions, rather than questions of an exploratory nature [18].

We conducted a literature review limited to articles published in English. We searched databases such as the Cochrane Library, Cinahl, Ovid Global Health, Ovid Medline, PubMed, Web of Science, Embase and Google Scholar, and performed a manual search of sources in articles’ reference lists. Grey literature was extracted manually through website search. However, some unpublished government documents (grey literature) in Portuguese were provided from Angola.

Our search was conducted in four steps. First, we searched maternal healthcare terms (obstetric delivery, pregnancy, childbirth, obstetric, birth, pregnancy, parturition, home childbirth, maternal death, reproductive health services, health provision). Then, we searched for terms related to the WHO six health system building blocks (health service, health workforce, health information, medical products, vaccine and technology, health finance, leadership and governance), and health service utilisation. Third, we searched for the names of the five countries (Angola, Eritrea, Ethiopia, Mozambique, and Rwanda). We combined the results of searching using these three sets of search terms. Finally, after screening the titles and then the abstracts, we included only the articles that met the framework criteria in the final step of our analysis.

## Results

We found a total of 1,176 articles of which 1,014 (86.2%) were peer-reviewed articles and 162 (13.8%) grey literature. Our final group of papers comprised 70 articles of which 51 (71.8%) were peer-reviewed articles and 19 (26.8%) grey literature (Figure 2). Table 1 shows the descriptive results of the demographic and health profile for each of the five countries that achieved ≥ 50% MMR reduction. Rwanda is the most densely populated country amongst the five countries (471 people per sq. Km), while Angola is the least densely populated (20 people per sq. Km). During 1990–2015, amongst all five countries, Ethiopia had the greatest  MMR reduction (71.8%) followed by Eritrea (68.5%).

**Table 1.** *Demographic and health profiles of sub-Saharan African countries that experienced war between 1990 and 2015 and reduced their maternal mortality ratios by more than 50%, 1990–2015.

	Angola	Eritrea	Ethiopia	Mozambique	Rwanda

Year of war	1975–2002	1961–1991; 1998–2000; 2004	1961–1991; 1998–2000; 2004	1977–1992	1994
					
Demographics, most recent years available					
Populations (million) (2015)	25	5	99	28	11
Rural population (2016)	55%	80%	80%	67%	71%
Population density per sq.-km of land area (2015)	20	47	99	36	471
Life expectancy (2015)	52	64	64	55	64
Gross Domestic Product (billion-US$) (2015)	102	3	61	15	8
Gross Domestic Product per capita (US$) (2015)	4,102	543 (2013)	619	525	697
Official development assistance per capita (US$) (2014)	10	28 (2011)	37	77	91
Health					
Maternal mortality ratios (per 100,000 live births), most recent years available					
1990	1,160	1,590	1,590	1,390	1,300
2015	477	501	353	489	290
MMR reduction during 1990–2015 (%)**	58.9	68.5	71.8	64.8	77.7
Health system factors					
Health services delivery					
Skilled birth attendants (% of utilisation) (2015)	50 (2007)	34 (2010)	15	54 (2011)	91
Antenatal care coverage at least four visits (% of utilisation) (2012)	47 (2009)	50	19	50	35
Health workforce					
Nurses and midwives’ density per 1,000 population	1.66 (2009)	0.58 (2004)	0.24 (2010)	0.41(2012)	0.69 (2010)
Physicians' density per 1,000 population	0.17 (2009)	0.05 (2004)	0.02 (2010)	0.04 (2012)	0.1 (210)
Healthcare financing					
Out–of–pocket health expenditure per capita (US$) (2014)	43	14	9	4	15
Health expenditure per capita (US$) (2014)	179	25	27	42	52
External resources for health (%)	3	28	42	49	46
Medical products and technologies					
Pharmaceutical personnel per 1,000 population	0.07 (2004)	0.03 (2004)	0.03 (2009)	0.06 (2012)	0.006 (2010)
Hospital beds (per 1,000 population)	0.8 (2005)	0.7 (2011)	6.3 (2011)	0.7 (2011)	1.6 (2007)

*Source of data based on the most current data available (unless otherwise indicated in brackets): All data were obtained from the world Bank.org unless otherwise stated; ANC data from UNICEF database (average of data from DHS, Multiple MICS, WHO, and UNICEF). ANC data for Angola from UN data. Health financing and pharmaceutical personnel data from WHO.

**To achieve MDG 5 (1990–2015), a country was required to reduce their MMR by more than 75% during 1990–2015.

**Figure 3.**
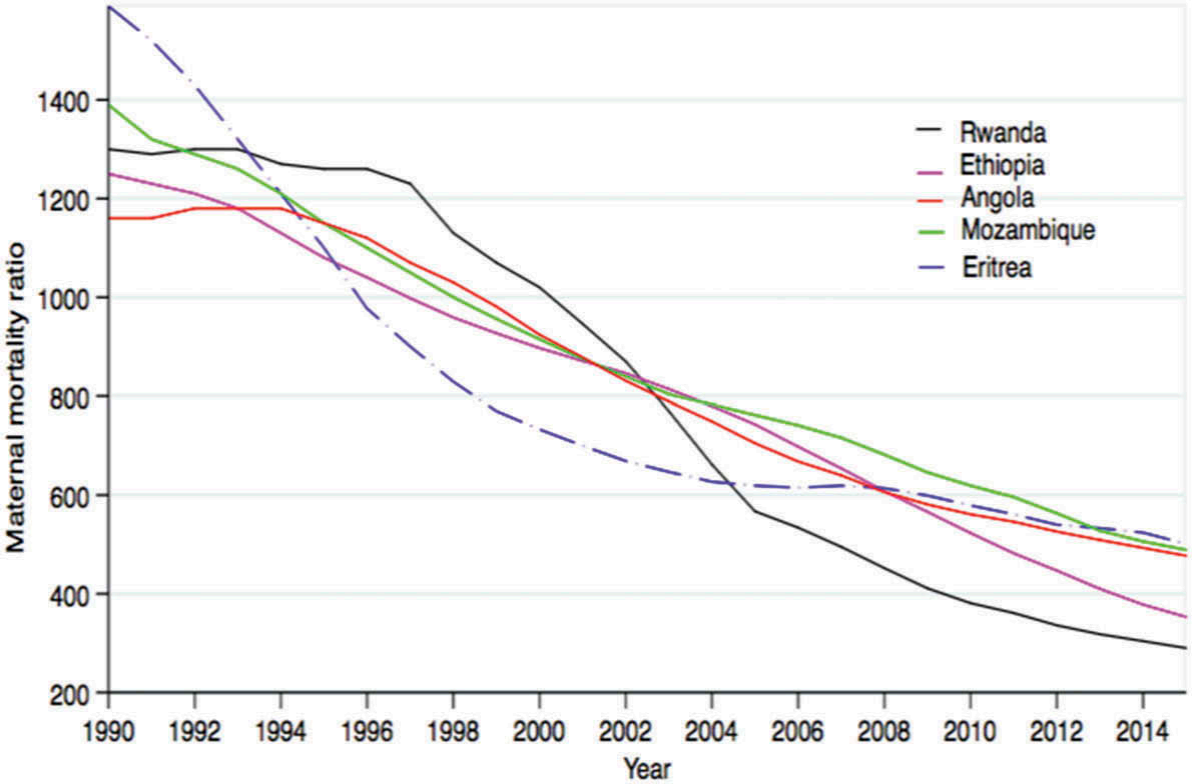
Maternal mortality decline in five sub-Saharan African countries affected by war during 1990–2015 (from the most significant decline in 2014). Data source: The 2015 Maternal Mortality Estimation Inter-Agency Group (MMEIG).

Table 2 shows the three areas of health system reforms common across all five countries that could explain MMR reduction equal to or greater than 50% as shown in Figure 3. These areas of reform were: (1) health systems decentralisation (related to Governance and Leadership WHO health system building block); (2) innovation related to the WHO workforce health system building block such as training of community healthcare workers (CHWs) (related to WHO workforce health system building block); and (3) health financing (related to WHO health system building financing block). All three findings contribute to health services delivery. However, our literature search did not reveal significant reforms concerning health information, essential medicine and geographical distance to healthcare services in any of the countries. Below we consider the three health system reforms that were common to the five countries we studied.

**Figure 4.**
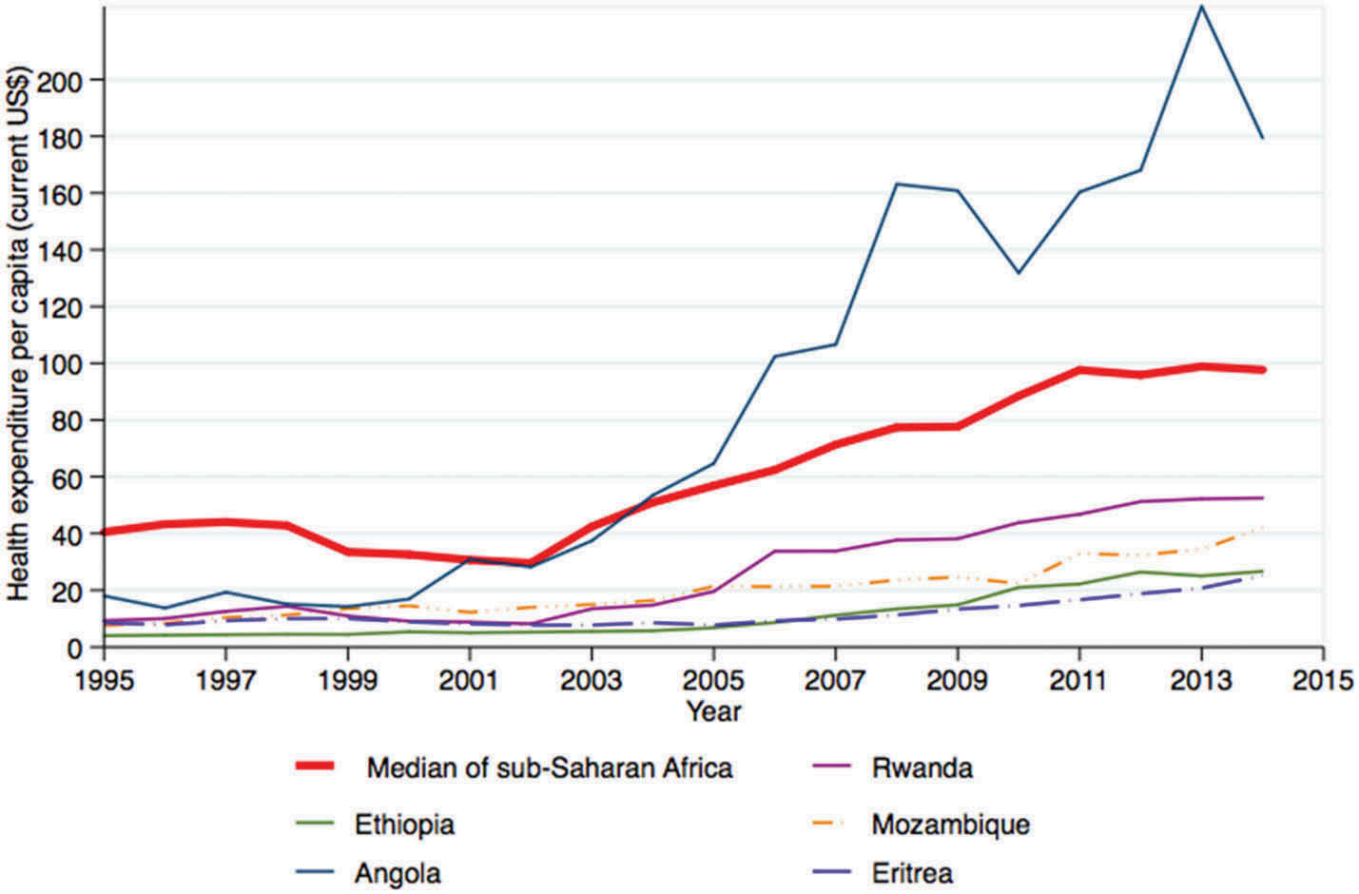
Total health expenditure in five "war-affected" countries compared to the median sub-Saharan region (most countries had no data prior to 1995). Data source: WHO

**Table 2.** Health system reforms with a significant effect on the provision of maternal health services in five sub-Saharan African countries, which experienced wars during 1990–2015, and achieved a significant maternal mortality reduction equal to or greater than 50% during the same period.

	Decentralisation	Healthcare workers (excluding volunteers)	Health-financing system
Angola	Implemented decentralisation in all 164 municipalities in 2010.	● Launched the Community Development and Health Agents programme in 2007.	● Receives only 14% of healthcare system funding from external donors.
		● Angola had 3,045 CHWs in 2014.● During 2005–2010, the number of doctors increased by 248% (849 vs 2956).● During 2005–2009, the number of nurses increased by 85% (16,037vs 29,592).● Contracting foreign doctors from Cuba.	● Out of pocket expenditure is 20%. The government contributes 80% of the total health expenditure.● Created independent financial units in 2008 in 68 municipalities and expanded in 2010 to include all 164 municipalities.
Eritrea	● Decentralisation with a varying degree. However, services such as maternal health services are fully decentralised and managed by the Zobas (regions).● Annual plans are developed by Zonal Healthcare units then submitted to the Ministry of Health.	● Established a Community Health Agents programme.● Eritrea had 800 CHWs in 2014.● Medical graduates are deployed to the regional areas for a period of one to two years.● Combined nursing and midwifery courses.● Contracting foreign doctors from China and Cuba.	● Developed fees retention policy for local health facilities in 2006.● By 2016, created a join pooling financial system for donor funding.● Eritrea introduced community health insurance, private health insurance, tax funds plus social health insurance and cost sharing.
Ethiopia	Decentralisation was outlined in the Health Policy Health Sector Development Programme IV 2010/11.	● Training of mid-level Health Extension Workers (HEWs) where two HEWs serve a kebele (village) of 3,000–5,000 population after a year of training.● Ethiopia had 34,000 CHWs in 2014.	● External donors funding contributes 40% of the health- financing system and 37% provided by households’ payments.● As an approach to Universal Health Coverage, since 2005, MoH developed a fees retention policy for local health facilities.● Ethiopia introduced Community-Based Health Insurance in 2008 and is currently piloted in various regions.
Mozambique	Decentralisation of management and tasks to the district directorates.	● Assistant medical officers (with obstetric surgical skills): currently, perform more than 90% of obstetric surgeries including caesarean sections in rural areas and 35% of emergency surgeries in urban areas.	● 50% aid dependent.● Created a joint pooling financial system for donor funding coordinated by a National provincial team.
		● Mozambique had 1,213 CHWs in 2014.	
Rwanda	Implemented the National policy on decentralisation in 2006 to strengthen community participation.	● Training of community health workers: three are elected by their community per village delegated to health promotion and maternity care––encouraging women to deliver in health facilities.● Rwanda had 45,000 CHWs in 2014.	● Implemented Community-Based Health Insurance in 2005 as an approach to the Universal Health Coverage covering 90% of the population, removing financial barriers to healthcare, therefore, increasing utilisation of services such as maternal health services. Non-covered services (10%) are paid by users but free for the poorest.
			● Introduced performance-based financing system in 2005, which gives incentive to health facilities to improve the quality of care in services such as antenatal care as a way to increase the number of facility-based deliveries.

### Health systems decentralisation

While the design of this study makes it difficult to link devolution of the health systems to MMR reduction directly, there were some noticeable governance reforms across all five countries that suggest that the decentralisation platform – the transfer of power, funding and decision-making to lower-level governments and communities – may have facilitated initiatives that contributed to the reduction in maternal mortality [15,19,20].

#### Angola

After experiencing a protracted civil war (1975–2000) just after its independence from Portugal, Angola implemented district-level fiscal decentralisation and a district health strategy to build municipalities’ capacity to improve primary healthcare services [21–23]. The plan was to create the Basic Law for the National Health Service through the decree n. 21-B/92 ruling that users should contribute reasonable fees to the public healthcare sector [24]. Under this law, health services were provided by the National Health Service, by the Military Health Services, and by the health services of large companies (public and private). These health services, in turn, were managed by the Ministry of Health, the Ministry of Defence and large corporations such as Chevron-Texaco [25]. By 2008, the government had implemented fiscal decentralisation in 68 out of all 164 municipalities nationwide, and by 2010 it was completed across the whole country [26].

These reforms aimed to address vulnerabilities in municipal health policy and healthcare provision in both the public and private sectors. Other efforts were made in 2010 by another presidential decree to improve the national health systems, through further regulation of infrastructure and health units [27,28].

#### Eritrea

Eritrea established a decentralisation policy before its independence from Ethiopia in 1991 by designing policies such as the Post-Independence Macro-Economic Policy Framework (MEPF) [29,30]. One aim of the MEPF was to improve access to healthcare facilities [29–31]. As a result of these significant policies such as the MEPF, the number of health facilities had increased by 170% by 2010 [32]. In 2012, maternal and other health services were decentralised to zoba (region) and sub-zoba levels [33]. Also, to ensure community participation, health policies are jointly reviewed annually by the Ministry of Health with the zoba health management teams [34].

#### Ethiopia

Decentralisation from the regional to the woreda (district) level was initiated in 2002 by implementing a flexible form of decentralisation in which power was devolved to ethnically diverse communities [20,35–38]. Each woreda has an administrative council composed of elected members [39,40]. The overall effectiveness of decentralisation to woreda level is suggested by an increase in contraception use by more than 200%, especially among the poor [37]. Ethiopia also implemented a series of health policies between 1997 and 2010 to decentralise healthcare [40–43]. For example, maternity care is now provided at three levels: health posts offer first aid, health centres provide primary essential maternity care, and district hospitals offer comprehensive emergency obstetric care [42].

#### Mozambique

Mozambique faced enormous challenges in rebuilding its healthcare systems following a protracted civil war that continued from 1975 to 1992 [44]. These challenges derived not only from the lack of health systems infrastructure, personnel and financing but also from the high burden of HIV, TB and malaria [44]. Nevertheless, and despite these challenges, Mozambique has undertaken steps to decentralise its health system. For example, in 1990 it introduced policies to devolve its health system to provinces, districts and then to autarquias (municipalities) [45–47]. However, while implementing decentralisation to the province level is seen as a sign of development, it remains a challenge [46,47].

#### Rwanda

Rwanda implemented decentralisation in 2000 as part of the government reconciliation process after the 1994 genocide [48,49]. Between 2005 and 2010 Rwanda designed the Twubakane ‘Let’s build together’ program to strengthen decentralisation of the health system at local government levels. Twubakane was undertaken with international partners and focussed on initiatives such as family planning and reproductive health in 12 out of the country’s 30 districts [48]. Twubakane resulted in the strengthening of the health system and improvement in health services [48]. By 2014, Rwanda had a network of 45,000 CHWs delivering decentralised health services in their communities as well as attending to essential maternal health needs – particularly important where access to health facilities is a barrier [50–56]. Rwanda also implemented partial fiscal decentralisation through Performance-Based Financing (PBF). While centrally managing maternal health service finances to prevent financial mismanagement, PBF provided incentives to staff and well-performing health facilities, allowing them to retain 40% of the revenues generated from both private and public healthcare sources [49,57,58].

### Innovation related to community healthcare workers (CHWs)

Boosting the number of healthcare workers is a standard feature of all the five war-affected countries. While they remain short of the recommended WHO requirement of 2.3 skilled healthcare workers (physicians and nurses/midwives) per 1000 population, all five countries have undertaken steps to address this particular problem [59]. For example, all five countries introduced an innovation related to the WHO workforce health system building block such as community healthcare workers programme [60]. Our study focuses on the paid "CHWs" cadre not volunteers.

#### Angola

Angola has the highest overall density of healthcare workers per 1,000 population of the five countries in this study, but the number of CHWs has been low [60]. In 2007, Angola introduced a training programme for CHWs, known as Community Development and Health Agents, and there were 3,045 CHWs in Angola in 2014 [60–62].

Other health reforms have led to increased numbers of other types of healthcare workers. For example, in 2005, the government introduced an administrative health reform linkage between the Ministry of Public Administration, Employment and Social Security, the Ministry of Finance, the provincial governments, and the central hospitals. This administrative health reform aimed to enable more reliable health services that offered adequate mobility and placement, doing so by providing both public and private institutions throughout the country [63,64]. In 2015, these institutions produced a total of 1547 graduates in medicine, medical sciences, and health studies, about 400 more than the previous year [65]. Also, between 2005 and 2010, Angola increased its number of doctors by 248% (from 849 to 2956) and the number of nurses increased by 85% (from 16,037 to 29,592) [66,67]. Angola also contracted approximately 1,500 Cuban-trained doctors [62,68]. However, despite the high health workforce numbers, as of 2016, half of the population in rural areas of Angola still had inadequate access to health services due to staff shortages [61,62].

#### Eritrea

Eritrea emphasised combining midwifery and nursing as a task-shifting strategy, as well as using foreign doctors from countries such as Cuba to deal with its health workforce shortage [29,69–71]. Consequently, between 2002 and 2012, the number of nurses/midwives increased by 48% (from 846 to 1,253) and associate nurses (diploma and certificate) increased by 70% (from 1,488 to 2,537) [29]. In 2005, Eritrea piloted a CHW program, and there were 800 CHWs, also known as community health agents, in the country by 2014 [60].

#### Ethiopia

In 2003, Ethiopia implemented the Health Extension Programme (HEP), managed by district governments [10,41]. As a result, overall health service delivery – measured by the number of people utilising health services – increased from 51% in 2000 to 92% in 2011[10,72]. The HEP policy was aimed at training CHWs to provide maternal health services in remote communities [73–77]. Each kebele (village) of 3,000–5,000 people is assigned two Health Extension Workers (HEWs) that have received a year of training [76–78]. In 2014, there were 34,000 HEWs with a ratio of 1:2,437 population (target: 1:2,500) [60,79]. HEWs are responsible for community mobilisation and deliver preventative maternity care services [76,77,80]. HEWs are required to spend 15% to 20% of their time at health posts and 75% to 80% of their time providing community outreach support [42,81]. Due to the critical shortage of physicians, Ethiopia also implemented task shifting by training of non-physician clinicians (NPCs) with skills needed for the provision of comprehensive emergency obstetric and neonatal care [82]. These NPCs were found to perform 63% of emergency procedures including caesarean sections [82].

#### Mozambique

Table 1 shows the size of the health workforce in Mozambique. Mozambique was one of the first countries in SSA to pioneer task shifting and training of healthcare workers including nurses, midwives, and CHWs (1,213 CHWs in 2014) to address health workforce shortages [60]. In 1978, Mozambique introduced the training program for CHWs who were known as Agentes Polivalentes Elementares (APEs) [83]. The task of these APEs was health promotion, including directing patients to the nearest health facility [83]. Another reform in Mozambique that was championed in 1984 by Pascoal Mocumbi (Minister of Health in Mozambique between 1980 and 1987) was the introduction of a category of healthcare workers with 2–3 years training specialising in obstetric surgery to address the shortage of doctors in rural areas [84,85]. By 2010, these healthcare workers were performing more than 90% of obstetric operations (including caesarean sections in rural areas) and 35% of emergency surgeries in urban areas, as well as helping pregnant women reach healthcare facilities [86–90].

#### Rwanda

In 2007, Rwanda introduced a CHWs’ initiative in each of its districts, involving three CHWs being elected by their community and providing preventive and curative services in their communities [52–54,56]. By 2014, there was a network of 45,000 CHWs in Rwanda implementing the decentralisation of health services by mobilising their communities, as well as attending to women’s urgent maternal needs – notably where access to health facilities was a barrier [50–56,60]. In addition to CHWs, Rwanda deployed more nurses, midwives and general doctors to the rural areas and trained traditional birth attendants [91,92].

### Health-financing system

State-sponsored public-financing systems play a significant role in shielding citizens from the catastrophic effects of out-of-pocket health expenditure – an expense that is a major barrier to increasing the number of facility-based deliveries, and improving utilisation of antenatal care, and other maternal health services [93–96]. As shown in Figure 4, health expenditure per capita in all countries, except Angola, is low compared to the average for SSA. We found the most current innovation in the state-sponsored public-financing system is in Rwanda, where Community-Based Health Insurance (CBHI) is more comprehensive than in the other four countries [21].

#### Angola

Health financing in Angola is less influenced by external donor funding than it is in the other four countries examined in this paper, with only 14% of Angola’s health funding being provided by aid [97]. The government contributes 80% of the total health expenditure, and out-of-pocket expenditure accounts for only 20% [21,88]. Twenty per cent of revenue is generated from private health insurance through the National Insurance Company of Angola, and the government provides the rest of the public funding [21].

#### Eritrea

Since 2006, Eritrea has attempted to enhance its financial pooling system and reduce the burden of out-of-pocket expenditure by increasing fees retention by healthcare facilities, as highlighted in the National Health Policy of 2010 [98,99]. Between 1988 and 2017, Eritrea introduced community health insurance and private health insurance as well as tax funds plus individual health insurance embedded within the public healthcare system, as by 2016 there were no privately owned clinics in the country [69,88,98,100,101]. However, there is no direct evidence as to the extent to which these initiatives impact on access to health services.

#### Ethiopia

Ethiopia has taken significant steps towards universal health coverage, although it is faced with the challenge of sustainability – donors contribute 40% of the health financing, while 37% is provided by householders’ payments [100,101]. Although out-of-pocket payments are still one of the barriers to accessing healthcare in the country, in 1998 Ethiopia started some decentralising reforms aimed at improving the health-financing system [96,102]. In 2006, Ethiopia initiated a woreda – based system that was financed by the government and development partners [37]. Between 2005 and 2008, the Ministry of Health developed a fees retention policy to provide revenues for local health facilities, as well as introducing the CBHI scheme in 2008 [94,102–104].

#### Mozambique

Mozambique receives some of the most substantial international donor funding in SSA, with 50% of the government funding depending on aid [87,105]. Due to the high level of donor funding, out-of-pocket health expenditure is minimum, and services are free for the poor, therefore enabling broader access to health services [106]. In 2000, the Ministry of Health and donors signed an agreement regarding coordination of aid to be channelled through the government financing system [87,107,108]. This agreement led to a decrease in vertical financing – funds directed towards specific interventions not fully integrated within the health system – from 82% in 2001 to 50% in 2007 [108,109]. In 2008, the government created a single financing pooling system and provincial coordination teams to coordinate donor funding [46,108].

Despite the numerous efforts by Mozambique to coordinate and direct donor funding towards building its local capacity, international donor funding remains under criticism for not contributing enough to strengthening the health system, for adding to dis-harmony in coordinating external resources, and for creating an unequal distribution of resources, thereby leading to disparities within the health system [109,110].

#### Rwanda

In Rwanda, two significant health-financing policies were introduced post-conflict: CBHI and PBF [49,57,111]. The introduction of CBHI led to a sharp increase in facility-based deliveries [112,113]. Rwanda piloted CBHI in 1999, and it was then fully implemented nationally in 2005 [54,88,114]. Fifty per cent of the CBHI funding comes from annual membership premiums, with the very poor exempt [115]. The national government funds cover the rest of the cost of CBHI from local and international organisations [116]. By 2015, CBHI comprised 90% of the population, removing financial barriers to accessing healthcare and therefore increasing utilisation of health services [114–116]. Non-covered services (10%) are paid for by users at the point of service but are free for the poorest [54,117]. CBHI currently covers facility-based delivery and provides caesarean sections free of charge [118].

Poor quality of care is one of the barriers to the utilisation of maternal health services [119,120]. In 2005, Rwanda initiated PBF as a form of financial incentive to improve the quality of care, subsequently leading to an increase in facility-based deliveries [112,121–123]. In addition to enhancing the quality of care, PBF also contributed to building local district capacity as a part of Rwanda’s decentralisation policy [123,124].

## Discussion

This study aimed to understand best health system practices in five war – affected SSA countries that experienced wars during 1990–2015 and yet managed to achieve a maternal mortality reduction – equal to or greater than 50% during the same period. Analysis of recent data from these five countries illustrates how some countries have used reforms to rebuild their health systems after the war. Taking MMR reduction as a sensitive indicator for the outcome of these reforms on health systems performance, three common reforms emerged as good practices: health systems decentralisation, health workforce innovations such as training of CHWs, and country’s ownership of health finance to minimise the influence of external donors on the provision of healthcare services.

One indicator of good governance is decentralisation, including decentralisation which facilitates the participation of sub-national stakeholders in decision-making [15]. SSA has seen progress in leadership to address its health challenges [125], and one example of such development is the varying degrees of decentralisation that have been achieved and have enabled the reform of healthcare systems [20,126]. Implementation of decentralisation requires political will. In all five countries that we studied, devolution was mainly driven by the political commitment of the same leaders or parties that were instrumental in ending the wars. This political commitment could be, at least in part, responsible for the success of decentralisation efforts in these countries, as it is implemented as part of a healing process towards reconciliation. The progress of decentralisation in the five countries also needs to be considered, particularly in relation to factors such as the duration of the war and the magnitude of any influence of the HIV epidemic. For example, Mozambique experienced both a lengthy war as well as the HIV/AIDS epidemic, which could have contributed to its relatively slow progress in the decentralisation of its health services and therefore slowed its MMR reduction [8].

Shortages in the healthcare workforce are another challenge facing most SSA countries. The current WHO recommendation requires a minimum of 4.45 doctors, nurses and midwives per 1,000 population to achieve the SDG goals [59]. Shortages in the healthcare workforce indicate that SSA countries, including the five countries examined in this study, need to employ more innovative approaches to increase their workforce. One such reform already in place is CHWs. All five countries use CHWs to address shortages in the health workforce and to improve access to health services in geographically isolated regions [18,96,127–130]. CHWs provide low-cost services and are easier to retain than doctors and nurses, especially in rural areas [131,132]. Despite CHWs playing an essential role in healthcare delivery, there are challenges related to absenteeism and perception of the services they provide as being of poor quality [37,133–137]. However, these challenges could be overcome by proper training and by offering incentives to reduce absenteeism.

This study is one of the very few studies to have examined health system reforms in war-affected SSA countries during 1990–2015 and their relationship to MMR reduction. However, our results are in line with the general findings described globally by other scholars [9,10,138,139] who investigated health system reforms in low-income countries, and how countries managed to deliver good healthcare with little resources. For example, like Balabanova et al., we found that an increase in the number of CHWs was one of the critical factors driving improvement in health systems performance in Ethiopia [9,10,138,139]. The important contribution of our study is the finding that factors identified by previous studies across multiple developing countries – particularly a study conducted by Pavignani and Colombo [140] about war-affected health systems – also apply in SSA countries affected by war. In their report, they analysed health services in Mozambique and Angola during 1975–2000 and concluded that health systems in war-affected regions should be a concern for the international community. Our study extends their work by analysing health systems in five countries.

Establishing causal links between health system reforms and outcomes is challenging – not least in a context of armed conflict where change can be rapid and unpredictable. While the lack of MMR reduction in the other six war-affected countries in SSA could be related to deficiencies in the three health system factors that we identified, lack of success could also be due to a combination of contextual circumstances that are peculiar to each country. Hence, this exploratory literature review is only able to identify relatively discrete reforms (hardware), with the understanding that there may be less tangible reasons for improved outcomes (software) [141]. Examples of such software include the strong determination for progress after the devastation of war; political deals between warring factions leading to decentralised governance; and significant amounts of foreign aid from strategic allies to support rebuilding efforts. Future research should seek to both disentangle and connect these hardware and software elements in real time, as countries undergo recovery and reform.

Although we conducted a broad literature review and consulted country experts, this study had some limitations. First, there was an imbalance in the number of published peer-reviewed articles among the five countries. Second, it was not possible to establish a link between decentralisation and health services delivery in Angola, Mozambique and Eritrea. Nevertheless, we have shown direct examples in both Rwanda and Ethiopia that suggest improvements in health systems performance associated with decentralisation. Third, it was difficult to get reliable data about the countries before their wars, which often dated back to the 1960s and 1970s. Fourth, while it is challenging to theorise and assume that the experiences of one country could be transferable to another, there is evidence to suggest that some reforms may be effectively employed across different countries. Two examples are the CHW reforms in SSA that started in Mozambique and the community-based health insurance schemes currently operating in Ethiopia and Rwanda (as well as in other SSA countries). Finally, we did not analyse war-affected SSA during 1990–2015 that did not achieve ≥ 50% MMR reduction. This means that we might have missed some valuable lessons that could be learned from those countries.

## Conclusion

Restoring health systems after disasters is an urgent concern, especially in SSA countries that have had wars. Reforms across all five countries that are examined in this study – Angola, Eritrea, Ethiopia, Mozambique, and Rwanda – were mainly driven by political commitment within each country to decentralise health systems and health workforce innovations such as CHWs’ programs. Our findings provide an insight from these countries which with further research could inform policy and thereby help other countries rebuild and maintain their health systems resilience, particularly in the aftermath of disasters such as wars. This is particularly relevant to countries emerging from conflict or instability including the Central African Republic, the Democratic Republic of Congo, and South Sudan. Further research on decentralisation models appropriate in different settings and their implementation challenges will be important, alongside governance research into how to bring women and children’s health to the fore amidst post-conflict policymaking.

## Paper context

Recently, there have been calls to strengthen health systems in sub-Saharan Africa. However, reforming those healthcare systems remains a challenge in some sub-Saharan African countries that have experienced wars. To our knowledge, this study is the first to explore a wide range of health system reforms in the five SSA countries that experienced wars during 1990–2015 and achieved ≥ 50% MMR reduction in the same period. Maternal mortality reduction is used as a sensitive measure of robustness of these countries’ health systems.
